# Allostatic (over) load measurement: Workflow and repository

**DOI:** 10.1371/journal.pone.0360639

**Published:** 2026-10-21

**Authors:** Shawna Beese, Jason Cross, David Rice, Trey L. DeJong

**Affiliations:** 1 College of Agricultural, Human, and Natural Resources Sciences, Washington State University, Pullman, Washington, United States of America; 2 CHORDS Lab, Washington State University, Bellingham, Washington, United States of America; 3 Center for Interdisciplinary Statistical Education and Research (CISER), Washington State University, Pullman, Washington, United States of America; Nanjing First Hospital, Nanjing Medical University, CHINA

## Abstract

Researchers have long studied allostatic (over)load as an estimated measure of individual cumulative stress over a lifetime. Often called the overall ‘wear and tear’ from social and environmental stressors, allostatic (over)load shows promise as a practical indicator of general health trends in community settings. This data processing workflow aims to document our overall approach and reasoning when calculating allostatic (over)load for data analysis and knowledge sharing. The included repository features an R script for generating datasets using this workflow from the following data sources: *All of Us* Research Program data repository. Health and Retirement Study (HRS). National Health and Nutrition Examination Survey (NHANES). Our allostatic (over)load measurement process, along with the linked repository, provides a reproducible workflow to process secondary data and offers insights into protocol-driven measurement practices in community environments.

## Introduction

Researchers have utilized allostatic (over)load as a conceptual model for the maladaptive biological processes that develop when our fight-or-flight acute stress response is activated as a chronic condition [1,2]. When detrimental environmental exposures, biological and psychosocial stressors, or other allostatic challenges are elevated, the hypothalamic–pituitary–adrenal (HPA) axis and sympathetic–adrenal–medullary (SAM) systems are activated with cascading biological responses as part of the acute stress response. If this acute stress response is perpetually activated due to continual perceived threat, maladaptation can occur over time to both the primary mediating pathways of the neuroendocrine and inflammatory response systems, but also the secondary mediating pathways of metabolic and cardiovascular responses [3].

For over two decades, researchers across various disciplines have also employed allostatic (over)load as an inferred measure of individual-level cumulative stress burden throughout the life course [3]. Often referred to as the overall ‘wear and tear’ from stressors and the resulting increase perceived stress [4,5], high allostatic load levels are a preclinical indicator of future chronic disease (e.g., cardiovascular disease, immune disorders, diabetes, anxiety, asthma) development as a result of the maladaptive responses that develop due to over taxation of the acute stress primary and secondary mediating pathways over time [6].

Since the original 10 biomarkers were first reported in the literature [7], several biomarker panels and statistical approaches have been proposed as a potential standardized method for calculating allostatic (over)load [8,9]. A comprehensive meta-analysis conducted in 2023 identifies a battery of five biomarkers that are most strongly associated with early indicators of generalized multi-system dysfunction resulting from the overburden of stress response among individuals in mid-life and beyond [10]. The five biomarkers index identified was based on the strength of association and biomarker availability. Since allostatic (over)load is a measure reflecting the cumulative burden of stress responses, we affirm the meta-analysis authors’ focus on mid-life and beyond to reach consensus on a generalized measure. Our team acknowledges that there is a demand for more statistically nuanced methods for measuring allostatic (over)load in research, especially when studying pathway-specific physiological processes [11,12]. However, as a research team interested in allostatic (over)load as a generalized outcome variable that predicts overall health and mortality risk, we find that the five-biomarker index articulated in McCrory et al. has practical applications for representing the cumulative individual-level multi-system stress response [10] for both data science and community research.

## Materials & methods

Although *All of Us*, HRS, and NHANES each support allostatic load research, biomarker availability, clinical context, and measurement frequency can differ. Although focusing on the five-biomarker index [10] allows for greater consistency across databases for calculating allostatic (over)load, distinctions between how each data source collects biomarker data must be carefully considered. With these concerns in mind, we provide consistent operating procedures across data sources to compute valid allostatic (over)load figures. That being said, we later recommend consideration of and sensitivity analysis relative to standardized clinical cutoffs for all biomarkers utilized.

Numerical analyses were conducted sequentially and independently for each data source (i.e., *All of Us*, HRS, and NHANES) using an identical analytic workflow implemented through dataset-specific R scripts with minor adaptations based on data source distinctions. No individual-level data were merged across sources. For each dataset, biomarker availability was assessed, preprocessing and exclusion criteria were applied, and allostatic load was constructed using internally derived risk thresholds. This approach enables independent replication by evaluating whether consistent methodological decisions yield convergent patterns across structurally distinct datasets, while avoiding assumptions required for pooled inference. Table 1 maps this analytic process for each dataset to specific R scripts and sections for reproducibility.

**Table 1:** Mapping of Allostatic (over)Load Analytic Workflow to Dataset-Specific R Scripts.

		*NIH All of Us*	*HRS*	*NHANES*
***Step***		***NIH_All_of_Us_dataset_construction.ipynb***	***HRS_dataset_construction.Rmd***	***NHANES_dataset_construction.Rmd***
***1***	Load data/ biomarker selection and filtering	Setup and Biomarker Extraction	~Line 68: Part 1 – Load, Clean, and Combine HRS Waves (2006–2016)	~Line 53: Part 1 – Download, Clean, and Combine NHANES Survey Cycles (1999–2023)
***2***	Data cleaning (invalid values, missingness, repeat measures)	Remove Duplicate Records	~Line 694: Handle HRS special codes …	~Line 987: Cycle-specific loading/cleaning sections; Combine 1999–2023 NHANES Data
***3***	Derived variables (WHtR, pulse, age)	Waist/Height Ratio and Heart Rate	~Line 745: Part 2 – Derive Biomarker Risk Indicators & Compute Allostatic Load	~Line 1000: Part 2 – Derive Biomarker Risk Indicators & Compute Allostatic Load
***4***	Plausibility filtering (NHANES-based)	Filter implausible [Biomarker] values	~Line 828: Remove biomarker values outside NHANES plausibility ranges	N/A
***5***	Biomarker completeness (biomarker_count)	Merge All Biomarkers into One Dataset (AL5)	~Line 886: Add biomarker count	~Line 1090: Compute biomarker_count
***6***	Quartile threshold calculation	Create Allostatic Load Scores: #calculate thresholds	~Line 833: Compute thresholds within each survey year	~Line 1105: Calculate Quartile Risk Thresholds
***7***	Risk indicator construction (0/1 per biomarker)	Create Allostatic Load Scores: #recode items to binary	~Line 849: Calculate risk scores	~Line 1153: Calculate Risk Scores
***8***	Allostatic load score (0–5)	Create Allostatic Load Scores: #sum for allostatic ordinal	~Line 861: Compute allostatic load score	~Line 1176: Calculate Allostatic (over)Load
***9***	Final dataset construction	Demographics and Final Preparation	~Line 922: Save and Export Prepared HRS Data	~Line 1198: Save and Export Prepared NHANES Data

The protocol described in this peer-reviewed article is published on protocols.io (https://dx.doi.org/10.17504/protocols.io.3byl4mq7zlo5/v1) and is included for printing purposes as S1 File.

Initially, our data processing approach began with an evaluation of the *All of Us* database for the study of perceived stress and three different methods of calculating allostatic (over)load [13]. Our refined data processing method for calculating allostatic (over)load, outlined in this document, initially utilized NIH *All of Us* Research Program (Table 2).

**Table 2:** Summary of Allostatic (over)Load Variable Calculation using NIH *All of Us* V.8.

Allostatic (over)load biomarkers	Variable name in All of Us	All of Us repository location
**C-reactive protein (CRP)** **-or-** **High Sensitivity** **C-reactive protein** **(HS-CRP)**	C reactive protein [Mass/volume] in Serum Plasma -or- C reactive protein [Mass/volume] in Serum Plasma by High sensitivity method	Electronic Health Record (EHR) • Labs & Measurements
Heart Rate*	Computed heart rate, mean heart rate of 2^nd^ and 3^rd^ measures	Measurements & Wearables • Physical Measurements
**High Density Lipoprotein (HDL)**	Cholesterol in HDL [Mass/volume] in Serum or Plasma	Electronic Health Record (EHR) • Labs & Measurements
**Glycated Hemoglobin (A1C)**	Hemoglobin A1c/Hemoglobin total in Blood	Electronic Health Record (EHR) • Labs & Measurements
Waist-to-Height Ratio (WHtR)*	Computed waist circumference, mean of closest two measures -and- Height	Measurements & Wearables • Physical Measurements

Note: *For all unique identifiers in NIH *All of Us,* physical measurement values are present; the completeness of laboratory biomarker data limited the total sample size to 37,866.

This procedure resulted in a sample of 37,866 unique participants (Supplementary Table 1 in S2 File) from the NIH *All of Us* respondents with version 8 (V.8) controlled tier data. The final data access date was in July 2025, and individual participants are only identified to the research team by their Participant Identification Number (PIN). The PIN was used to merge fragmented data frames into a single, complete dataset. According to the *All of Us* data documentation, two standard operating procedures were used for collecting physical measurements [14].

### Summary of physical measurements calculation

- Heart Rate [15]

- Mean of the 2nd and 3rd measures in the case of repeated measures.

- Waist-to-Height Ratio (WHtR)

- This variable was calculated by first determining the calculated waist circumference [16], and then dividing it by the calculated height [17].
- The mean of the two closest measures is used in the case of repeated measures.

### Summary of biomarkers data cleaning and processing

The following data processing approach (Steps 1–4) was used for the two physical measurement biomarkers, and the three laboratory biomarkers: a) High-density Lipoprotein (HDL), b) Glycated Hemoglobin (A1C), and c) C-reactive protein (CRP).

1 ***Ensure only comparable lab measurements were used.***
  a Of all the different lab tests that could be used to represent each of the laboratory biomarkers, we used ONLY the most common test with established acceptance in the literature (e.g., serum or plasma test on HDL; Hemoglobin A1c/Hemoglobin total in blood).
  b The one exception to the above was that both serum or plasma CRP and serum or plasma high-sensitivity C-reactive protein (HS-CRP) are established in the literature. We cleaned the data for participants who had either-but kept the values sorted with separate CRP and HS-CRP columns. If a participant had values from both, we only used their CRP in the allostatic (over)load calculation. This procedure was used to ensure no duplicate weighing of CRP values for any given participant. CRP is preferred over HS-CRP because it also has a clinical threshold established in the literature (Table 3). The CRP clinical threshold cannot be used for HS-CRP.

**Table 3:** Clinical Thresholds for Potential Sensitivity Analysis.

Allostatic (over)load biomarkers and clinical threshold	Citation
**Pulse or Heart rate (HR): ≥ 90 beats/min**	**Parker et al., 2022 [18] • Borell et al., 2020 [19] • Howard & Sparks, 2016 [20] • Levine & Crimmins, 2014 [21]
**High-Density Lipoprotein (HDL): < 40 mg/dl**	**Parker et al., 2022 [18] • Akinyemiju et al., 2020 [22] • Borell et al., 2020 [19] • Howard & Sparks, 2016 [20] • Levine & Crimmins, 2014 [21]
**C-Reactive Protein (CRP): ≥ 3.0 mg/L**	**Parker et al., 2022 [18] • Akinyemiju et al., 2020 [22] • Borell et al., 2020 [19] • Howard & Sparks, 2016 [20] • Levine & Crimmins, 2014 [21]
**Glycated Hemoglobin (A1C): ≥ 6.4%**	**Parker et al., 2022 [18] • Borell et al., 2020 [19] • Howard & Sparks, 2016 [20] • Levine & Crimmins, 2014 [21]
**Waist-to-Height Ration (WHtR): > 0.5**	Browning et al., 2010 [23]
* Waist circumference (WC): ≥ 102 cm (male)/ ≥ 88 cm (female)	Seo et al., 2017 [24]

Note: *In addition to waist-to-height ratio as the preferred anthropometric measure [10], we additionally provide a clinical threshold for waist circumference. **Parker et al.[18], published these meta-analysis clinical cutoffs.

2 ***Determine if there were repeated measures.***
  a Some individual respondents had multiple observations recorded on different dates (repeated measures). To establish a single metric for each individual, the mean was calculated over the recorded metrics for an individual within one year from the most recent measurement date**.**
  b Additionally, the observation was removed if only NA values were recorded for the individual.

3 ***Removal of implausible values.***
  a While statistically based outlier cutoffs were considered for the biomarkers (i.e., four standard deviations (*SD*) from the mean or four median absolute deviations (*MAD*) from the median), the severely skewed nature of some biomarkers made application of these methods impossible to apply consistently for all data sources. The former method retained many values likely to be viewed as implausible in the *All of Us* database, while the latter method truncated concerning amounts of datapoints when applied to HRS and NHANES datasets (see Table 4). Therefore, to minimize the influence of implausible values while retaining biologically meaningful extremes, boundaries based on biologically plausible ranges are an attractive alternative.
  b Given that no such biological boundaries are well established in the literature, we utilized absolute minimum and maximum values across all years for each biomarker from the NHANES database (Table 5). NHANES is a nationally representative survey with a multi-staged clustered data collection and rigorous laboratory quality control and, as such, provides empirically grounded limits for biologically plausible values. These bounds should be broadly generalizable as NHANES is designed to represent the non-institutionalized U.S. population using probability sampling: CRP = 0.08 to 296 mg/L, A1C = 2.0 to 18.8%, HDL = 5.0 to 226.0 mg/dL, Pulse = 0.0 to 224.0 bpm, and WHtR = 0.24 to 1.14. These thresholds were intentionally conservative to avoid excluding rare but valid observations documented in a high-quality population-based dataset rather than imposing artificially restrictive thresholds. Biomarker distributions following application of the NHANES-based plausibility filters are summarized in Table 6.

**Table 4:** Comparison of Biomarker Filtering Methods Across Datasets.

*Biomarker*	*Dataset*	*% ± NHANES Abs. Min/Max*	*% ± 4 SD*	*% ± 4 MAD*
***A1C***	NIH	0.126	0.111	13.508
	HRS	0.003	1.150	8.126
	NHANES	*N/A*	1.451	8.000
***CRP***	NIH	1.368	0.064	21.852
	HRS	0.687	0.893	14.400
	NHANES	*N/A*	0.945	14.710
***HDL***	NIH	0.045	0.004	3.047
	HRS	0.003	0.132	2.083
	NHANES	*N/A*	0.276	2.908
***PULSE***	NIH	0.000	0.064	1.169
	HRS	0.000	0.151	1.476
	NHANES	*N/A*	0.094	1.155
***WHtR***	NIH	0.144	0.110	1.683
	HRS	0.112	0.045	2.406
	NHANES	*N/A*	0.078	1.142

**Table 5:** NHANES-Referenced Biomarker Plausibility Ranges.

*Biomarker*	*Min*	*Max*
***A1C***	2.00	18.80
***CRP***	0.08	296.00
***HDL***	5.00	226.00
***PULSE***	0.00	224.00
***WHtR***	0.24	1.14

**Table 6:** Biomarker Distribution Summary Across NIH All of Us, HRS, and NHANES.

*Biomarker*	*NIH All of Us* *(n = 37,866)*	*HRS* *(n = 32,906)*	*NHANES* *(n = 61,610)*
***A1C***	6.08 (1.34)	5.91 (1.05)	5.60 (0.99)
***CRP***	18.40 (37.09)	3.95 (7.40)	3.76 (7.81)
***HDL***	53.44 (17.25)	54.99 (16.58)	52.95 (15.32)
***Pulse***	73.05 (12.82)	70.18 (11.55)	72.98 (12.37)
***WHtR***	0.61 (0.10)	0.62 (0.09)	0.57 (0.10)

Note: Values are presented as mean (standard deviation).

4 ***Calculate the allostatic (over)load risk category***
  a The top quartile CRP, heart rate, A1C, and WHtR were coded as 1 = high risk.
  b The bottom quartile of HDL was coded 1 = high risk, as HDL is a cardio-protective biomarker.
  c All observations determined to be in the lower 75%-tiles of risk were coded 0 = normal risk.

Each participant is assigned a cumulative score of 0–5 (with 0 being the lowest and 5 being the highest calculated risk) based on the sum of their high-risk scores across all five biomarkers. Although we acknowledge that statistical modeling power is lost by dichotomizing each biomarker into high-normal risk, this is the most utilized method and the method presented in the consensus article [10]. All biomarkers must be present to calculate a respondent’s overall score.

### Recommendations for clinical cutoffs

The majority of studies in extant allostatic (over) load literature have used quartiles to calculate the sample specific relative risk for each biomarker [11]. This makes sense because until recently, allostatic (over)load measurement has largely been the domain of researchers. As the utility of this measure is studied and quantified for use in clinical and community practice, a body of scientific literature is needed to bridge the gap between sample-specific relative risk and the application of established, clinically relevant cutoffs (absolute risk) for biomarkers. Our team recommends and plans to use clinical cutoffs established in the literature to determine high and normal-risk categories. Additionally, we recommend reporting findings from both methods as part of a sensitivity analysis in future research. To calculate allostatic (over)load, use the same schema as above (step 4).

### HRS and NHANES datasets

Allostatic (over)load is calculated for the Health and Retirement Study (HRS; Table 7; Supplementary Table 2 in S2 File) and National Health and Nutrition Examination Survey (NHANES; Table 8; Supplementary Table 3 in S2 File) with the same approach as described for the NIH *All of Us*, with minor adaptations.

**Table 7:** Summary of Allostatic (over)Load Variable Calculation using Health and Retirement Survey (HRS).

	Biomarker Data	HRS CORE Data
**2016**	High-Density Lipoprotein (HDL) Glycated Hemoglobin (A1C) C-reactive protein (CRP)	Pulse (heart rate) Waist-to-height ratio (WHtR) Demographics (age, sex at birth, race, ethnicity)
**2014**	High-Density Lipoprotein (HDL) Glycated Hemoglobin (A1C) C-reactive protein (CRP)	Pulse (heart rate) Waist-to-height ratio (WHtR) Demographics (age, sex at birth, race, ethnicity)
**2012**	High-Density Lipoprotein (HDL) Glycated Hemoglobin (A1C) C-reactive protein (CRP)	Pulse (heart rate) Waist-to-height ratio (WHtR) Demographics (age, sex at birth, race, ethnicity)
**2010**	High-Density Lipoprotein (HDL) Glycated Hemoglobin (A1C) C-reactive protein (CRP)	Pulse (heart rate) Waist-to-height ratio (WHtR) Demographics (age, sex at birth, race, ethnicity)
**2008**	High-Density Lipoprotein (HDL) Glycated Hemoglobin (A1C) C-reactive protein (CRP)	Pulse (heart rate) Waist-to-height ratio (WHtR) Demographics (age, sex at birth, race, ethnicity)
**2006**	High-Density Lipoprotein (HDL) Glycated Hemoglobin (A1C) C-reactive protein (CRP)	Pulse (heart rate) Waist-to-height ratio (WHtR) Demographics (age, sex at birth, race, ethnicity)

Note: HDL = High-density lipoprotein; A1C = Glycated hemoglobin; CRP = C-reactive protein.

**Table 8:** Summary of Allostatic (over)Load Variable Calculation using National Health and Nutrition Examination Survey (NHANES).

	C-Reactive Protein/ Variable Name	Glycated Hemoglobin/ Variable Name	High-Density Lipoprotein/ Variable Name	Pulse/ Variable Name	Waist to Height Ratio/ Variable Name	DEMOS/ Variables Names
**2021-2023**	LBXHSCRP	LBXGH	LBDHDD	BPXOPLS3	BMXWAIST BMXHT	SEQN RIAGENDR RIDAGEYR RIDRETH3 & 1
**2017–2020** **(Pre-Pandemic)**	LBXHSCRP	LBXGH	LBDHDD	BPXOPLS3	BMXWAIST BMXHT	SEQN RIAGENDR RIDAGEYR RIDRETH3 & 1
**2017-2018**	LBXHSCRP	LBXGH	LBDHDD	BPXPLS or BPXOPLS3	BMXWAIST BMXHT	SEQN RIAGENDR RIDAGEYR RIDRETH3 & 1
**2015-2016**	LBXHSCRP	LBXGH	LBDHDD	BPXPLS	BMXWAIST BMXHT	SEQN RIAGENDR RIDAGEYR RIDRETH3 & 1
**2013-2014**	–	LBXGH	LBDHDD	BPXPLS	BMXWAIST BMXHT	SEQN RIAGENDR RIDAGEYR RIDRETH3 & 1
**2011-2012**	–	LBXGH	LBDHDD	BPXPLS	BMXWAIST BMXHT	SEQN RIAGENDR RIDAGEYR RIDRETH3 & 1
**2009-2010**	LBXCRP	LBXGH	LBDHDD	BPXPLS	BMXWAIST BMXHT	SEQN RIAGENDR RIDAGEYR RIDRETH1
**2007-2008**	LBXCRP	LBXGH	LBDHDD	BPXPLS	BMXWAIST BMXHT	SEQN RIAGENDR RIDAGEYR RIDRETH1
**2005-2006**	LBXCRP	LBXGH	LBDHDD	BPXPLS	BMXWAIST BMXHT	SEQN RIAGENDR RIDAGEYR RIDRETH1
**2003-2004**	LBXCRP	LBXGH	LBXHDD	BPXPLS	BMXWAIST BMXHT	SEQN RIAGENDR RIDAGEYR RIDRETH1
**2001-2002**	LBXCRP	LBXGH	LBDHDL	BPXPLS	BMXWAIST BMXHT	SEQN RIAGENDR RIDAGEYR RIDRETH1
**1999-2000**	LBXCRP	LBXGH	LBDHDL	BPXPLS	BMXWAIST BMXHT	SEQN RIAGENDR RIDAGEYR RIDRETH1

Note: LBXCRP = C-reactive protein(mg/dL); LBXHSCRP = HS C-Reactive Protein (mg/L); LBXGH = Glycohemoglobin(%); LB2HDL = HDL-cholesterol (mg/dL); LBDHDD = Direct HDL-Cholesterol (mg/dL); LBDHDL = HDL-cholesterol (mg/dL); LBXHDD = Direct HDL-Cholesterol (mg/dL); BPXPLS = 60 sec. pulse (30 sec. pulse * 2); BPXOPLS3 = Pulse – 3rd oscillometric reading; BMXWAIST = Waist Circumference (cm); BMXHT = Standing Height (cm); SEQN = Respondent sequence number; RIAGENDR = Gender; RIDAGEYR = Age in years at screening; RIDRETH1 = Race/Hispanic origin; RIDRETH3 = Race/Hispanic origin w/ NH Asian.

The final data access date was in June 2025 for HRS and July 2025 for NHANES. All individual participants were deidentified to the research team, with individual patient identification codes assigned by both secondary data sources. The minor adaptations in the data processing workflow are:

1. Pulse (heart rate) as a single data point is used, as opposed to a mean of repeated measures for Allostatic (over)Load calculation with the Health and Retirement Study (HRS) and National Health and Nutrition Examination Survey (NHANES) as a data source.
2. Only C-reactive protein (CRP) is used with the Health and Retirement Study (HRS). Both CRP and high-sensitivity C-reactive protein (HS-CRP), depending on availability, are used with the National Health and Nutrition Examination Survey (NHANES) as a data source.

To assess whether dataset selection led to variations in the five biomarkers assessed and their cutoffs, descriptives for all five biomarkers in each dataset are presented in Table 6. We also assessed cross-dataset biomarker cutoff variability using the coefficient of variation of dataset-specific risk quartile cutoffs to allow for comparison without pooling individual-level data. These variations were assessed both within data sources but across years and averaged across years and between data sources. These coefficients of variation suggest no heterogeneity between the datasets and are also presented in Table 9.

**Table 9:** Cross-Dataset Variability of Biomarker Risk Thresholds.

*Biomarker*	*Risk Direction*	*NIH All of Us*	*HRS*	*NHANES*	*SD*	*CV*
***A1C***	≥ 75th percentile	6.27	6.13	5.72	0.2850	0.0472
***CRP***	≥ 75th percentile	14.20	4.29	4.14	5.7632	0.7638
***HDL***	≤ 25th percentile	41.00	42.66	42.00	0.8347	0.0199
***Pulse***	≥ 75th percentile	81.00	77.50	82.00	2.3629	0.0295
***WHtR***	≥ 75th percentile	0.674	0.669	0.622	0.0291	0.0444

Note: Values represent average quartile-based risk thresholds calculated within each dataset. Risk direction indicates whether higher (≥75th percentile) or lower (≤25th percentile) values correspond to increased allostatic load. SD and CV (coefficient of variation) summarize variability in cutpoints across data sources.

To respect the potential for bias in allostatic (over)load calculations, we present crosstabulations in Table 10 to consider whether distributions of allostatic load totals and allostatic (over)load differ depending on which database is being utilized. A χ^2^ test of independence was conducted to assess whether an association exists between data source and allostatic load distribution. Results of the analysis showed that a statistically significant association exists (χ^2^(10) = 307.95, p = < 0.001, Cramer’s v = 0.034 [95% CI: 0.031, 0.038]). Although these findings suggest significant variation in the distribution of allostatic load between data sources, the size of this association is small when considering the degrees of freedom and conventional heuristics of interpretation. Distributions of allostatic load were therefore rather consistent between data sources with a small amount of variation.

**Table 10:** Distribution of Allostatic (over)Load Scores Across Data Sources.

*AL Score*	*NIH All of Us* *(n = 37,866)*		*HRS* *(n = 32,906)*		*NHANES* *(n = 61,610)*	
	**n**	**%**	**n**	**%**	**n**	**%**
***0***	12,608	33.30	10,075	30.62	19,154	31.09
***1***	11,729	30.98	10,715	32.56	18,878	30.64
***2***	7,751	20.47	7,068	21.48	12,359	20.06
***3***	4,074	10.76	3,601	10.94	7,554	12.26
***4***	1,438	3.80	1,231	3.74	3,004	4.88
***5***	266	0.70	216	0.66	661	1.07
***Total***	**37,866**	**100**	**32,906**	**100**	**61,610**	**100**

Note: Values are presented as frequency (n) and row percentage (%). Sample sizes reflect complete cases only (i.e., observations with all five biomarkers available).

### Community collected biomarker measurements

When collecting allostatic (over)load metrics as primary sourced data in community settings, we recommend contracting with an appropriately credentialed lab for the collection of:

- High-density Lipoprotein (HDL)
- Glycated Hemoglobin (A1C)
- C-reactive protein (CRP)

Physical measurements will likely be collected independently at the community site by internal staff. We present the following PhenX protocols, as the PhenX Toolkit aims to provide widely accepted, standardized measurement procedures across various research domains [25].

- Resting Heart Rate [26]
- Waist-to-Height Ratio (WHtR)
  - This variable will be computed by first calculating the waist circumference [27] and then dividing it by the calculated standing height [28].

Data collected in a community setting cannot compete with the database sources discussed in this paper in terms of sample size, so the stabilizing effects of large samples will be less reliable in the community data context. As such, we recommend conducting sensitivity analysis to compare clinical cutoffs for the community data, the larger database cutoffs described here, and the clinical cutoffs determined from the literature summarized in Table 3. In this way, community researchers can validate their data for allostatic (over)load calculation. Additionally, the discrete nature of allostatic load (0–5) and allostatic (over)load typically require statistical methods that assume minimum frequencies of each value. As such, minimum samples to be collected in a community setting should aim to meet threshold requirements for all allostatic load values (e.g., expected frequency > 5 for all values in a χ^2^ test).

### Limitations and future research implications

For allostatic load research among the data sources reviewed here, there is a historical lack of consistency for how allostatic load is specified. Duong et al., for example, found that among 21 published NHANES studies, 21 distinct specifications of allostatic load were utilized [9]. The promise of a unifying framework for specifying load in McCrory et al. can help to establish rigor for researchers using these databases. Although ALI-5 is strongly evidence based, its uptake for research in these contexts is still emergent [10]. Beese et al. found that for the *All of Us* database, the ALI-5 allostatic load specification is markedly more feasible for conducting research relative to other common specifications of allostatic load [13]. The workflow presented here aims to increase the uptake of the ALI-5 framework for research focusing on allostatic load. Additionally, prior studies in this context have typically focused on utilization of a single data source – most often NHANES [9] or HRS [29] – with biomarker selection and allostatic load specifications being determined within each dataset. The current workflow establishes a consistent, cross-cohort workflow that works across three large databases through a modular, reproducible R-based pipeline that preserves dataset independence while standardizing analytical logic. We aimed to assist researchers focused on population health who have faced recurring challenges in harmonizing biomarker definitions, documenting analytical decisions, and assessing comparability without pooling individual-level data by explicitly mapping conceptual scoring decisions to dataset-specific coding. This should help to establish consistency and replicability in the field of allostatic load research which has historically lacked these qualities and exhibited vast heterogeneity in its research infrastructure.

A limitation of this approach is that it is grounded in research that focused explicitly on mid-life and beyond. Future synthesis (e.g., meta-analysis) research into the temporal variations of allostatic (over)load across the life course is still warranted.

While plausibility for biomarker value was determined in the current study based on minima and maxima from the NHANES database, future research should work to establish biologically plausible ranges for these biomarkers to aid researchers in ensuring consistency and generalizability even when data collection efforts are more localized and limited in scope relative to the databases discussed here. The issues we encountered when attempting to apply consistent, statistically based outlier detection methods (i.e., retention of likely implausible data and exclusion of likely valid data) demonstrate the clear need for well-established ranges of plausibility.

A perennial critique of allostatic (over)load calculation is that it is dichotomized into a high-risk/low-risk schema. A dichotomized schema has been used for decades and has been justified in the literature.^10(p.6, para3)^ However, we do recommend that future research use sensitivity analyses with established clinical thresholds. Also, if future researchers develop other categorical sub-classifications (e.g., low 0–2; moderate 3; high 4–5), sensitivity analysis with the classic method is also recommended, as this would establish a comparative context to practices already established in the literature.

## Conclusion

The data processing workflow, along with our linked repository, are aimed to provide a transparent and reproducible method by which to calculate allostatic (over)load. Additionally, we are sharing our process as a resource to encourage discourse with other researchers and data scientists who are also studying allostatic (over)load as a generalized variable to infer preclinical maladaptive responses to stressors that often precede the development of future chronic diseases.

## Supporting information

S1 File Step-by-step protocol, also available on protocols.io. (PDF)

S2 File Supplementary Tables. (PDF)

## References

[1] Juster R-P, Misiak B. Advancing the allostatic load model: From theory to therapy. Psychoneuroendocrinology. 2023;154:106289. doi: 10.1016/j.psyneuen.2023.106289 37202225

[2] McEwen BS. Allostasis and allostatic load: Implications for neuropsychopharmacology. Stress and the Brain. 2013;:2–18. 10.1016/S0893-133X(99)00129-3 10649824

[3] Beckie TM. A systematic review of allostatic load, health, and health disparities. Biol Res Nurs. 2012;14(4):311–46. doi: 10.1177/1099800412455688 23007870

[4] Barboza Solís C, Fantin R, Kelly-Irving M, Delpierre C. Physiological wear-and-tear and later subjective health in mid-life: Findings from the 1958 British birth cohort. Psychoneuroendocrinology. 2016;74:24–33. doi: 10.1016/j.psyneuen.2016.08.018 27567118

[5] McEwen BS, Seeman T. Protective and damaging effects of mediators of stress. Elaborating and testing the concepts of allostasis and allostatic load. Ann N Y Acad Sci. 1999;896:30–47. doi: 10.1111/j.1749-6632.1999.tb08103.x 10681886

[6] McEwen BS, Lasley EN. The end of stress as we know it. Joseph Henry Press. 2002.

[7] Seeman TE, Singer BH, Rowe JW, Horwitz RI, McEwen BS. Price of adaptation--allostatic load and its health consequences. MacArthur studies of successful aging. Arch Intern Med. 1997;157(19):2259–68. 9343003

[8] Beese S, Postma J, Graves JM. Allostatic load measurement: A systematic review of reviews, database inventory, and considerations for neighborhood research. Int J Environ Res Public Health. 2022;19(24):17006. doi: 10.3390/ijerph192417006 36554888 PMC9779615

[9] Duong MT, Bingham BA, Aldana PC, Chung ST, Sumner AE. Variation in the calculation of allostatic load score: 21 examples from NHANES. Journal of Racial and Ethnic Health Disparities. 2017;4(3):455–61. 10.1007/s40615-016-0246-8 PMC5195908 27352114

[10] McCrory C, McLoughlin S, Layte R, NiCheallaigh C, O’Halloran AM, Barros H, et al. Towards a consensus definition of allostatic load: A multi-cohort, multi-system, multi-biomarker individual participant data (IPD) meta-analysis. Psychoneuroendocrinology. 2023;153:106117. doi: 10.1016/j.psyneuen.2023.106117 37100008 PMC10620736

[11] Carbone JT, Clift J, Alexander N. Measuring allostatic load: Approaches and limitations to algorithm creation. J Psychosom Res. 2022;163:111050. doi: 10.1016/j.jpsychores.2022.111050 36228435

[12] Liu SH, Juster R-P, Dams-O’Connor K, Spicer J. Allostatic load scoring using item response theory. Compr Psychoneuroendocrinol. 2020;5:100025. doi: 10.1016/j.cpnec.2020.100025 35754455 PMC9216382

[13] Beese S, Abshire DA, DeJong TL, Carbone JT. An evaluation of the All of Us Research Program database to examine cumulative stress. J Am Med Inform Assoc. 2024;31(12):2968–73. doi: 10.1093/jamia/ocae201 39058629 PMC11631057

[14] Introduction to All of Us Physical Measurement Data Collection and Transformation Methods. https://support.researchallofus.org/hc/en-us/articles/29888188023060-Introduction-to-All-of-Us-Physical-Measurement-Data-Collection-and-Transformation-Methods. Accessed 2025 August 13.

[15] Standard Operating Procedure - Heart Rate Measurement. https://support.researchallofus.org/hc/en-us/article_attachments/30122059316628. Accessed 2025 August 13.

[16] Standard Operating Procedure - Waist-Hip Circumference Measurement. https://support.researchallofus.org/hc/en-us/article_attachments/30122059296404. Accessed 2025 August 13.

[17] Standard Operating Procedure - Height and Weight Measurement. https://support.researchallofus.org/hc/en-us/article_attachments/30122059308052. Accessed 2025 August 13.

[18] Parker HW, Abreu AM, Sullivan MC, Vadiveloo MK. Allostatic load and mortality: A systematic review and meta-analysis. Am J Prev Med. 2022;63(1):131–40. doi: 10.1016/j.amepre.2022.02.003 35393143

[19] Borrell LN, Rodríguez-Álvarez E, Dallo FJ. Racial/ethnic inequities in the associations of allostatic load with all-cause and cardiovascular-specific mortality risk in U.S. adults. PLoS One. 2020;15(2):e0228336. doi: 10.1371/journal.pone.0228336 32053626 PMC7018050

[20] Howard JT, Sparks PJ. The effects of allostatic load on racial/ethnic mortality differences in the United States. Popul Res Policy Rev. 2016;35(4):421–43. doi: 10.1007/s11113-016-9382-4

[21] Levine ME, Crimmins EM. A comparison of methods for assessing mortality risk. Am J Hum Biol. 2014;26(6):768–76. doi: 10.1002/ajhb.22595 25088793 PMC4286244

[22] Akinyemiju T, Wilson LE, Deveaux A, Aslibekyan S, Cushman M, Gilchrist S, et al. Association of allostatic load with all-cause andcancer mortality by race and body mass index in theREGARDS cohort. Cancers (Basel). 2020;12(6):1695. doi: 10.3390/cancers12061695 32604717 PMC7352652

[23] Browning LM, Hsieh SD, Ashwell M. A systematic review of waist-to-height ratio as a screening tool for the prediction of cardiovascular disease and diabetes: 0·5 could be a suitable global boundary value. Nutr Res Rev. 2010;23(2):247–69. doi: 10.1017/S0954422410000144 20819243

[24] Seo D-C, Choe S, Torabi MR. Is waist circumference ≥102/88cm better than body mass index ≥30 to predict hypertension and diabetes development regardless of gender, age group, and race/ethnicity? Meta-analysis. Prev Med. 2017;97:100–8. doi: 10.1016/j.ypmed.2017.01.012 28137662

[25] Cox LA, Hwang S, Haines J, Ramos EM, McCarty CA, Marazita ML, et al. Using the PhenX toolkit to select standard measurement protocols for your research study. Curr Protoc. 2021;1(5):e149. doi: 10.1002/cpz1.149 34038028 PMC8251725

[26] Protocol - cardiorespiratory fitness - non-exercise test estimate. https://www.phenxtoolkit.org/protocols/view/151401. Accessed 2025 August 13.

[27] Protocol - waist circumference - waist circumference *NHANES*. [cited 2025 Aug13]. Available from: https://www.phenxtoolkit.org/protocols/view/21604

[28] Height - Standing Height. https://www.phenxtoolkit.org/protocols/view/20706. Accessed 2025 August 13.

[29] Pak T-Y, Kim G. Association of food insecurity with allostatic load among older adults in the US. JAMA Netw Open. 2021;4(12):e2137503. doi: 10.1001/jamanetworkopen.2021.37503 34874403 PMC8652609

