# Supplementary material for: Allostatic (over) load measurement: Workflow and repository
Source: PLoS One. 2026 Oct 21;21(10):e0360639. doi: 10.1371/journal.pone.0360639 (PMC13686455; doi:10.1371/journal.pone.0360639)
Supplement: Supplementary file 1 [file pone.0360639.s001.pdf]

Jul 28, 2026

Protocol for Calculating Allostatic (over)Load from *All of Us*, HRS, and NHANES Data

DOI
https://dx.doi.org/10.17504/protocols.io.3byl4mq7zlo5/v1

Shawna Beese[1,2], Jason Cross[1,2], David Rice[3], Trey DeJong[2,3]

[1]College of Agricultural, Human, and Natural Resources Sciences, Washington State University, Pullman, WA, USA;
[2]CHORDS Lab, Washington State University, Bellingham, WA, USA;
[3]Center for Interdisciplinary Statistical Education and Research (CISER), Washington State University, Pullman, WA, USA

Extension.CHORDSLab

Create & collaborate more with a free account

Edit and publish protocols, collaborate in communities, share insights through comments, and track progress with run records.

Create free account

OPEN ACCESS

DOI: https://dx.doi.org/10.17504/protocols.io.3byl4mq7zlo5/v1

External link: https://github.com/CHORDSLab/Allostatic-over-Load-Repository

Protocol Citation:
Shawna Beese, Jason Cross, David Rice, Trey DeJong 2026. Protocol for Calculating Allostatic (over)Load from All of Us, HRS, and NHANES Data. **protocols.io**
https://dx.doi.org/10.17504/protocols.io.3byl4mq7zlo5/v1

Manuscript citation:
Beese, S., Cross, J., Rice, D., DeJong, T. (2025). Allostatic (over)Load Measurement: Workflow and repository. [In Peer Review] medRxiv 2025.07.31.25332519; DOI: **doi.org/10.1101/2025.07.31.25332519**

License: This is an open access protocol distributed under the terms of the **Creative Commons Attribution License**, which permits unrestricted use, distribution, and reproduction in any medium, provided the original author and source are credited

Protocol status: Working
We use this protocol and it's working

Created: July 23, 2026

Last Modified: July 28, 2026

Protocol Integer ID: 321465

Keywords: Allostatic load, NHANES, HRS, All of Us, Biomarkers, Secondary data analysis, R, measure of individual cumulative stress, us research program data repository health, individual cumulative stress, practical indicator of general health trend, measurement practices in community environment, repository health, following data source, environmental stressor, us research program data, general health trend, health, national health, nhanes data abstract, retirement study, nutrition examination survey, calculating allostatic, data source, data processing workflow, hr, reproducible workflow, data analysis

Abstract

Abstract

Researchers have long studied allostatic (over)load as an estimated measure of individual cumulative stress over a lifetime. Often called the overall ‘wear and tear’ from social and environmental stressors, allostatic (over)load shows promise as a practical indicator of general health trends in community settings. This data processing workflow aims to document our overall approach and reasoning when calculating allostatic (over)load for data analysis and knowledge sharing. The included repository features an R script for generating datasets using this workflow from the following data sources:

- *All of Us* Research Program data repository
- Health and Retirement Study (HRS)
- National Health and Nutrition Examination Survey (NHANES)

Our allostatic (over)load measurement process, along with the linked repository, provides a reproducible workflow to process secondary data and offers insights into protocol-driven measurement practices in community environments.

Attachments

DOCX

Protocol Tables.docx

29KB

Guidelines

- Dataset-specific implementation details are available in the linked repository
- Table 5 maps each workflow step to corresponding R script sections
- Different datasets may require dataset-specific variable harmonization

Materials

Data Sources

- All of Us
- HRS
- NHANES

Software

- R
- Relevant R packages

Procedure

An overview of the allostatic (over)load workflow and its mapping to the dataset-specific R scripts is provided in Table 5.

Before start

- Obtain approved access to relevant datasets.
- Install R and required packages.
- Download the workflow repository.
- Familiarize yourself with dataset-specific variable names.

1 ***Select and extract biomarkers***

Allostatic load as defined by the ALI-5 framework requires the extraction of variables to identify quantitative measures of C-reactive protein (CRP), heart rate, high density lipoprotein (HDL), glycated hemoglobin (A1C), and waist-to-height ratio (WHtR). The names and locations of relevant biomarker variables for *All of Us* (Table 1), HRS (Table 3), and NHANES (Table 4) are summarized in the accompanying tables and implemented in the linked repository. Additionally, all data collection waves of interest should be merged into a single dataset while retaining information regarding the wave source for each datapoint.

2 ***Clean data***

Identify invalid values, missingness, and repeated measures among the extracted biomarkers. Specific techniques are shown in dataset-specific R scripts.

3 ***Create derived variables***

For certain data sources, variables such as WHtR, pulse, and age must be derived for proper calculation of allostatic (over)load. The techniques used by our lab are detailed in respective R scripts.

4 ***Apply plausibility filters***

Cases which do not meet the NHANES-based plausibility filter should be considered for removal to ensure data robustness. This must only be applied to the All of Us and HRS datasets. The boundaries for this filter are defined as the absolute minimum and maximum values across all NHANES survey years and are summarized in Table 6.

5 ***Calculate biomarker completeness***

Calculation of biomarker counts helps to ensure unbiased ALI-5 totals by ensuring that all observations include information for all five biomarkers. Cases which do not include data for all five biomarkers should be excluded from ALI-5 analysis dataset.

6 ***Calculate quartile thresholds***

ALI-5 totals are based on whether an observation lies within the risk threshold for each biomarker. Specifically, CRP, heart rate, A1C, and WHtR used the top quartile, and HDL used the bottom quartile for identifying biomarker risk. In this step, high-risk quartile thresholds are identified either within each dataset or using the clinical thresholds summarized in Table 2. R scripts are provided by data source.

7 ***Create binary risk indicators***

Based on risk quartiles identified in step 6, all observations should be dichotomized based on whether they meet the risk threshold for each biomarker (1=high risk; 0=normal risk).

8 ***Calculate allostatic load score***

Binary biomarker risk indicators created in step 7 should be summed for all cases with complete data for all 5 biomarkers (identified in step 5). The resulting score ranges from 0 to 5, with higher scores indicating greater allostatic (over)load. This total score can also be dichotomized to represent high and normal risk allostatic load scores which is common among allostatic load research (e.g., 0-2 normal; 3-5 high).

9 ***Export final dataset***

The total allostatic load score and, if applicable, allostatic (over)load value should be exported along with any other variables of interest in your study. A unique identifier should also be retained to ensure data integrity and flexibility.

Acknowledgements

The authors would like to extend deep gratitude to the Data 424: Data Analytics Capstone Team 2 members—Mariah Bergquist, Maya Dietrich, William Ripplinger, and Owen Williams—for sharing their technical skills and expertise in the development of the National Health and Nutrition Examination Survey (NHANES) dataset. We also want to thank lab member Kristen Barta for her final proofreading of this manuscript.

We want to express our appreciation to all participants in the NIH *All of Us* Research Program, the Health and Retirement Study (HRS), and the National Health and Nutrition Examination Survey (NHANES). Only through their contributions was this study made possible.
