# Supplementary material for: Allostatic (over) load measurement: Workflow and repository
Source: PLoS One. 2026 Oct 21;21(10):e0360639. doi: 10.1371/journal.pone.0360639 (PMC13686455; doi:10.1371/journal.pone.0360639)
Supplement: Supplementary file 2 [file pone.0360639.s002.pdf]

Supplementary Table 1. Demographic Characteristics for Allostatic (over)Load Sample from NIH *All of Us* V.8

	Female (N=22,513)	***Male (N=14,902)***	***Overall (N=37,866)***
Race			
American Indian or Alaska Native	209 (0.9%)	195 (1.3%)	411 (1.1%)
Asian	412 (1.8%)	250 (1.7%)	669 (1.8%)
Black or African American	4,392 (19.5%)	2,214 (14.9%)	6,710 (17.7%)
Middle Eastern or North African	111 (0.5%)	89 (0.6%)	200 (0.5%)
More than one population	1,032 (4.6%)	552 (3.7%)	1,607 (4.2%)
None indicated, none of these, skip	3,388 (15%)	1,826 (12.2%)	5,307 (14%)
White	12,955 (57.5%)	9,757 (65.5%)	22,929 (60.6%)
Ethnicity			
Hispanic or Latino	3,298 (14.6%)	1,640 (11.0%)	4,973 (13.1%)
Not Hispanic or Latino	18,604 (82.6%)	12,768 (85.7%)	31,725 (83.8%)
Prefer not to answer, skip, none of these	611 (2.7%)	494 (3.3%)	1,168 (3.1%)
Age			
Mean (SD)	55.6 (14.5)	59.9 (13.8)	57.3 (14.4)
Median [Min, Max]	57.0 [<18, 103]	61.4 [<18, 100]	58.8 [<18, 103]

Note: Not all cross-tabs add up to 100% due to the masking of cells with n < 20; and n = 451 respondents identifying as a sex at birth other than Female or Male. All ages under 18 years are reported as <18 for the min.

Supplementary Table 2. Demographic Characteristics for Allostatic (over)Load Sample from Health and Retirement Survey (HRS)

	2006 (N=4,658)	*2008 (N=3,442)*	*2010 (N=6,857)*	*2012 (N=6,306)*	*2014 (N=5,940)*	*2016 (N=5,703)*	*Overall (N=32,906)*
Sex							
Female	2759 (59.2%)	2045 (59.4%)	3905 (56.9%)	3600 (57.1%)	3579 (60.3%)	3310 (58.0%)	19198 (58.3%)
Male	1899 (40.8%)	1397 (40.6%)	2952 (43.1%)	2705 (42.9%)	2361 (39.7%)	2393 (42.0%)	13707 (41.7%)
Missing	0 (0%)	0 (0%)	0 (0%)	1 (0.0%)	0 (0%)	0 (0%)	1 (0.0%)
Race							
White/Caucasian	3885 (83.4%)	32 (0.9%)	996 (14.5%)	85 (1.3%)	41 (0.7%)	706 (12.4%)	5745 (17.5%)
Black/African American	554 (11.9%)	9 (0.3%)	596 (8.7%)	28 (0.4%)	22 (0.4%)	307 (5.4%)	1516 (4.6%)
Other (incl. Native/Asian/PI)	206 (4.4%)	5 (0.1%)	285 (4.2%)	28 (0.4%)	5 (0.1%)	56 (1.0%)	585 (1.8%)
Don't Know/NA	11 (0.2%)	0 (0%)	9 (0.1%)	1 (0.0%)	0 (0%)	8 (0.1%)	29 (0.1%)
Refused	2 (0.0%)	0 (0%)	2 (0.0%)	0 (0%)	0 (0%)	0 (0%)	4 (0.0%)
Missing	0 (0%)	3396 (98.7%)	4969 (72.5%)	6164 (97.7%)	5872 (98.9%)	4626 (81.1%)	25027 (76.1%)
Ethnicity							
Mexican American/Chicano	238 (5.1%)	6 (0.2%)	248 (3.6%)	18 (0.3%)	2 (0.0%)	150 (2.6%)	662 (2.0%)
Other Latino/a (incl. Puerto Rican, Cuban)	144 (3.1%)	0 (0%)	177 (2.6%)	15 (0.2%)	6 (0.1%)	125 (2.2%)	467 (1.4%)
Refused	1 (0.0%)	0 (0%)	0 (0%)	0 (0%)	0 (0%)	0 (0%)	1 (0.0%)
Missing	4275 (91.8%)	3436 (99.8%)	6432 (93.8%)	6273 (99.5%)	5932 (99.9%)	5428 (95.2%)	31776 (96.6%)
Age							
Mean (SD)	67.3 (10.6)	69.5 (10.2)	66.2 (10.8)	67.0 (10.6)	68.0 (10.9)	65.4 (11.6)	67.1 (10.9)
Median [Min, Max]	67.0 [30.0, 99.0]	70.0 [27.0, 108]	65.0 [49.0, 99.0]	65.0 [49.0, 100]	66.0 [19.0, 103]	64.0 [25.0, 102]	66.0 [19.0, 108]

Note: Data source Health and Retirement Survey (HRS) core data (publicly reported) and biomarker data (requires access to sensitive data; permissions required).

Supplementary Table 3. Demographic Characteristics for Allostatic (over)Load Sample from National Health and Nutrition Examination Survey (NHANES)

	1999-2000 (N=5,978)	2001-2002 (N=6,394)	2003-2004 (N=6,088)	2005-2006 (N=6,098)	2007-2008 (N=5,929)	2009-2010 (N=6,371)	2015-2016 (N=5,805)	2017-2018 (N=5,400)	2017-2020 (Pre-Pandemic) (N=7,554)	2021-2023 (N=5,993)	Overall (N=61,610)
Sex											
Female	3,081 (51.5%)	3,306 (51.7%)	3,047 (50.0%)	3,132 (51.4%)	2,966 (50.0%)	3,196 (50.2%)	2,958 (51.0%)	2,736 (50.7%)	3,782 (50.1%)	3,229 (53.9%)	31,433 (51.0%)
Male	2,897 (48.5%)	3,088 (48.3%)	3,041 (50.0%)	2,966 (48.6%)	2,963 (50.0%)	3,175 (49.8%)	2,847 (49.0%)	2,664 (49.3%)	3,772 (49.9%)	2,764 (46.1%)	30,177 (49.0%)
Race											
Mexican American	2,004 (33.5%)	1,579 (24.7%)	1,434 (23.6%)	1,486 (24.4%)	1,118 (18.9%)	1,319 (20.7%)	1,091 (18.8%)	789 (14.6%)	1,001 (13.3%)	477 (8.0%)	12,298 (20.0%)
Non-Hispanic Black	1,246 (20.8%)	1,396 (21.8%)	1,483 (24.4%)	1,545 (25.3%)	1,180 (19.9%)	1,101 (17.3%)	1,184 (20.4%)	1,203 (22.3%)	1,833 (24.3%)	684 (11.4%)	12,855 (20.9%)
Non-Hispanic White	2,180 (36.5%)	2,926 (45.8%)	2,733 (44.9%)	2,632 (43.2%)	2,694 (45.4%)	2,940 (46.1%)	1,891 (32.6%)	1,875 (34.7%)	2,653 (35.1%)	3,441 (57.4%)	25,965 (42.1%)
Other Hispanic	352 (5.9%)	272 (4.3%)	193 (3.2%)	181 (3.0%)	698 (11.8%)	660 (10.4%)	774 (13.3%)	480 (8.9%)	750 (9.9%)	671 (11.2%)	5,031 (8.2%)
Other Race (Including Multi-Racial)	196 (3.3%)	221 (3.5%)	245 (4.0%)	254 (4.2%)	239 (4.0%)	351 (5.5%)	865 (14.9%)	1,053 (19.5%)	1,317 (17.4%)	720 (12.0%)	5,461 (8.9%)
Age											
Mean (SD)	37.8 (22.2)	37.5 (21.6)	38.8 (22.6)	37.8 (21.6)	44.7 (20.7)	43.4 (20.5)	43.2 (20.4)	45.2 (20.8)	44.6 (20.4)	48.4 (20.6)	42.1 (21.5)
Median [Min, Max]	32.0 [12.0, 85.0]	33.0 [12.0, 85.0]	34.0 [12.0, 85.0]	33.0 [12.0, 85.0]	44.0 [12.0, 80.0]	43.0 [12.0, 80.0]	43.0 [12.0, 80.0]	46.0 [12.0, 80.0]	45.0 [12.0, 80.0]	52.0 [12.0, 80.0]	41.0 [12.0, 85.0]

Note: Data source used only publicly reported NHANES data. Age (RIDAGEYR) was top-coded at 85 years for 1999–2006 and at 80 years for 2007–2023. Because complete-case allostatic (over)load scoring required CRP measurements, NHANES survey cycles 2011–2012 and 2013–2014 were not included in the analytic sample due to CRP unavailability.
